# Effect of sodium substitution by yttrium on the structural, dielectric and electrical properties of Ba_2_Na_(1-3x)_Y_x_Nb_5_O_15_ ceramics

**DOI:** 10.1016/j.heliyon.2023.e21037

**Published:** 2023-10-17

**Authors:** El Hassan Yahakoub, Amine Bendahhou, Ilyas Jalafi, Fatima Chaou, Soufian EL Barkany, Zahra Bahari, Mohamed Abou-Salama

**Affiliations:** a Laboratory of Molecular Chemistry, Materials and Environment, Department of Chemistry, Faculty Multidisciplinary Nador, University Mohamed Premier, B.P. 300, Selouane, Nador 62700, Morocco

**Keywords:** X-ray diffraction, Tungsten bronze, Dielectric, Ferroelectric and Nyquist plot

## Abstract

The effects of Na^+^ substitution by Y^3+^ on the structural, microstructural, dielectric and electrical properties of Ba_2_Na_(1-3x)_Y_x_Nb_5_O_15_ compositions with (x = 0, 0.02 and 0.04) have been studied in detail. The solid solutions of different compositions were prepared by the solid state reaction route method and characterized by X-Ray Diffraction (XRD), Scanning Electron Microscopy (SEM), and Complex Impedance Spectroscopy (CIS) techniques. The XRD study confirmed that all prepared compositions have a single-phase orthorhombic tungsten bronze structure with space group *Cmm*2 at room temperature. The microstructural studies revealed a grain shape and size change in response to increasing Y^3+^ concentration. The dielectric properties of the obtained compositions are evaluated over a temperature range of 40–600 °C. The dielectric properties were improved for the Y_2_O_3_-substituted Ba_2_NaNb_5_O_15_ compound compared to the undoped Ba_2_NaNb_5_O_15_ compound. The non-Debye type relaxation mechanism is confirmed by the -Z″ versus Z′ traces. The grain contribution was studied using an equivalent electrical circuit with a Resistor R, a Capacitor C, and a Constant-Phase Element CPE in parallel, in the absence of the grain boundary response and the electrode effect in the frequency range 10 Hz-1MHz. The experimental AC conductivity data were evaluated by using Jonscher's power law. The activation energies obtained from the relaxation and conduction processes, present two different regions as a function of temperature related to the two electrical processes for the prepared ceramics.

## Highlights

• The phase structure of Ba_2_NaNb_5_O_15_ ceramics is modified by Y_2_O_3_ doping.
• A strong decrease in the electrical conductivity of Y_2_O_3_-doped ceramics compared to BNN.
• Single and doubly ionized oxygen vacancies are responsible for electrical conduction processes in Ba_2_Na_(1-3x)_Y_x_Nb_5_O_15_ ceramics.

## 1 Introduction

**Fig. .1:**
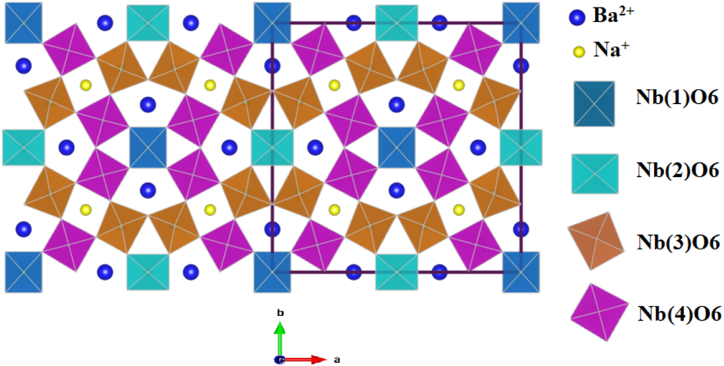
Crystal structure of the BNN sample drawn with the software "VESTA" in *Cmm*2.

Ferroelectric materials have increasingly been used in modern electronics sectors due to their numerous applications, such as piezoelectric, optoelectric, nonlinear optical, and pyroelectric devices [[1], [2], [3]]. Pb-free ferroelectric materials have captured the interest of the scientific and commercial sectors, owing to the severity of environmental concerns surrounding the use of lead [2,4,5]. Over the past few years, researchers have conducted numerous extensive studies on lead-free ferroelectric ceramics of perovskite structure ABO_3_, with particular attention to BaTiO_3_ and Bi_0.5_Na_0.5_TiO_3_-based ceramics as relaxor ferroelectrics [[6], [7], [8], [9], [10]]. Although some research has been dedicated to developing lead-free dielectric capacitors with different crystal structures, limited attention has been given to exploring other promising lead-free dielectric capacitors for energy storage applications. Further investigation is needed to identify these materials. As the second most important dielectric material for the perovskite type, lead-free Tungsten Bronze (TB) is known for its complex and intriguing structure. This dielectric material, similar to perovskites, comprises deformed BO_6_ octahedra connected by corners, creating three distinct sites: triangular, square and pentagonal. The chemical formula can be written as (A2)_2_(A1)C_2_B_5_O_15_, where the A1 and A2 (pentagonal and square sites respectively) may be occupied by all or part of a larger cation such as Sr^2+^, Ba^2+^, Ca^2+^, Na^+^, or K^+^, B-site is filled by a high valence cation such as W^6+^, Nb^5+^ or Ti^4+^, and the C site is usually vacant due to the gap being too small [[11], [12], [13], [14], [15], [16], [17], [18], [19], [20], [21]]. Among the tungsten bronze systems, the compound Ba_2_NaNb_5_O_15_ (BNN) has recently received much attention [12,13,22,23]. The structural, dielectric and electrical properties of Ba_2_NaNb_5_O_15_ ceramics have been extensively studied [12,24,25]. Note that in this structure, the pentagonal sites are occupied by Ba^2+^ ions, the square sites by Na^+^ (and induce an intense deformation), while the triangular tunnel remains empty. Niobium ions Nb^5+^ are statistically distributed in site B to form Nb(1)O_6_, Nb(2)O_6_, Nb(3)O_6_ and Nb(4)O_6_ octahedra, as shown in Fig. 1 [12,23,26].

Regarding the structural properties of this compound, BNN is a uniaxial material, it shows a series of phase transitions: orthorhombic (ferroelectroelastic) to tetragonal (ferroelectric) at about 300 °C, tetragonal (ferroelectric) to tetragonal (paraelectric) at T_c_ = 580 °C; on the other hand, its room temperature space group is *Cmm*2 [12,27]. The ferroelastic structure (*Cmm*2) was symmetry at room temperature as confirmed by *Jamieson* and *Niizeki* et al. [28,29]. The modification of the structural, dielectric and electric properties of the TB ceramics by the introduction of elements with strong charges like Bi^3+^, Nd^3+^,Y^3+^, … that replace the elements with weak charges like Na^+^ and K^+^ in the A1 and A2 sites have been widely reported [22,[30], [31], [32]]. Finlay *D. Morrison* et al. reported that the Curie temperature (T_c_) associated with the tetragonal (ferroelectric) to tetragonal (paraelectric) transformation in the compound Ba_4_R_0.67_Nb_10_O_30_ (where R=La^3+^, Nd^3+^, Sm^3+^, Gd^3+^, Dy^3+^, and Y^3+^) decreases as the rare-earth ionic radius increase, resulting in a decrease in the tetragonal distortion [33]. In addition, tungsten bronze ceramics' dielectric and electrical performance are highly dependent on their crystal structure and morphology [14,34,35]. For example, *liangliang Liu* et al. have found that the grain size distribution dominated the ferroelectric nature of KSr_2_Nb_5_O_15_ tungsten bronze (KSN). In other words, the dielectric anomalies are not only controlled by the compositions, but also by the microstructures of the ceramics [14]. The ceramic type consists of conductive grains separated by insulating grain boundaries, as described by the Maxwell-Wagner model. At low frequencies, the high value of ε′ can be attributed to factors such as grain boundary effects, oxygen vacancies, interfacial dislocations and charged defects. However, as the frequency increases, the decrease in ε′ is due to the fact that all species contributing to the polarization lag behind the external field [36]. Research on the characterization of Ba_2_Na_(1-3x)_(A^3+^)_x_Nb_5_O_15_ compounds (with A^3+^ high valence ions) has remained so far insufficient, either XRD or by CIA. In this work, doping with high valence ions changes the concentration of oxygen vacancies in our material, whereby Y^3+^ ions partially replace Na^+^ ions in the BNN compound. The structure, microstructure, dielectric and electrical properties of BNN and Y^3+^ doped BNN samples can be studied to find a relationship between these properties.

## 2 Experimental procedure

### 2.1 Materials and methods

Ceramics with compositions $\text{Ba}{\text{Na}}_{\left(1-3x\right)}Y_{x}{\text{Nb}}_{5}O_{15}$ (x = 0.00, 0.02, and 0.04) (denoted as BNN, BNYN0.02, and BNYN0.04, respectively) were synthesized by a high temperature solid-state reaction method. The high purity starting materials are: Na_2_CO_3_, BaCO_3_, Y_2_O_3_ and Nb_2_O_5_ (Sigma-Aldrich 99 %). All starting materials were preheated to 300 °C for one day (24 h), then weighed in stoichiometric amounts and mixed well by ethanol milling in the presence of zirconia balls for 2h. Then, the mixed powders were dried overnight at 80 °C, homogenized in an agate mortar for 30 min, and then calcined at 1200 °C for 6 h in the air. After grinding and drying, the undoped and Y^3+^ doped BNN ceramics were grounded again with the addition of an appropriate amount of polyvinyl alcohol (PVA) as a binder, and then uniaxially pressed (20 KN) in the mold (2 mm thickness and 12 mm diameter). Finally, the formed pellets were sintered in air at 1300 °C (6h). The temperature was increased to 700 °C with a rate of 10 °C.min^−1^, after a plateau of 1 h at this temperature and with a speed of 5 °C.min^−1^ the temperature rises to the sintering temperature, followed by a second 6 h plateau at T = 1300 °C and allowed to cool to room temperature. The surfaces of the pellets were properly polished to obtain conductive electrodes for measuring dielectric and electrical properties. A silver layer was used on the top and bottom surfaces of the pellets to serve as a parallel plate.

### 2.2 Characterization

The Crystal structure identification of BNN, BNYN0.02, and BNYN0.04 powders was performed by XRD. The instrument used was a D2 PHASER diffractometer equipped with a copper anode (CuKα_1_ et CuKα_2_). The diffractograms were recorded at 293 K (0.01° steps, 5°–70° 2θ range). To have both surfaces of the sintered pellets flat and parallel, it is necessary to go through a polishing step with emery paper, and then heat treated at 1300 °C (30 min). The surface morphology of BNN, BNYN0.02 and BNYN0.04 ceramics sintered at 1300 °C was observed by SEM (TESCAN VEGA III LM). Image J software was used to estimate the average grain size from the SEM images. The sintered ceramics were coated with silver on both sides, then annealed at 300 °C (30 min) to make the silver adhere to the sample, before analyzing its dielectric and electrical properties, using a bioLogic impedance analyzer (MTZ-35) equipped with a temperature controller, both room temperature and high temperature impedance measurements were performed. Electrical and dielectric measurements were performed in the temperature range of 40 °C–600 °C and at frequencies from 1Hz to 1 MHz.

## 3 Results and discussion

### 3.1 Crystal structure

**Fig. .2:**
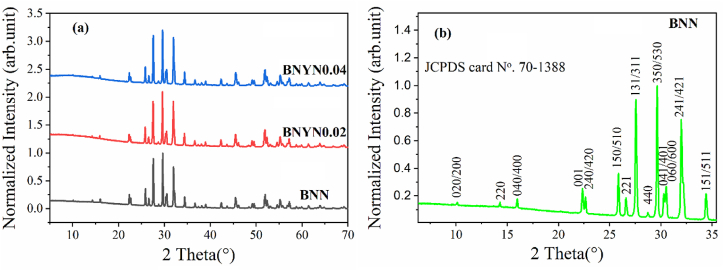
(a) XRD diffractograms of BNN, BNYN0.02 and BNYN0.04 powders calcined at 1200 °C and (b) the magnification of the BNN composition in the range of 05°–35°.

The X-ray diffraction spectra recorded at room temperature of the different compounds Ba_2_Na_(1-3x)_Y_x_Nb_5_O_15_ as (x = 0, 0.02 and 0.04) are presented in Fig. 2(a). Note that a first indexation was performed for these compositions using the computer program Full prof. The XRD spectra of the ceramics are in agreement with the standard JCPDS card N^o^. 70–1388 [37]. The crystallographic planes (hkl) corresponding to the standard JCPDS card N^o^. 70–1388 in the range 2θ 5°–35° are shown in Fig. 2(b).

**Fig. 3:**
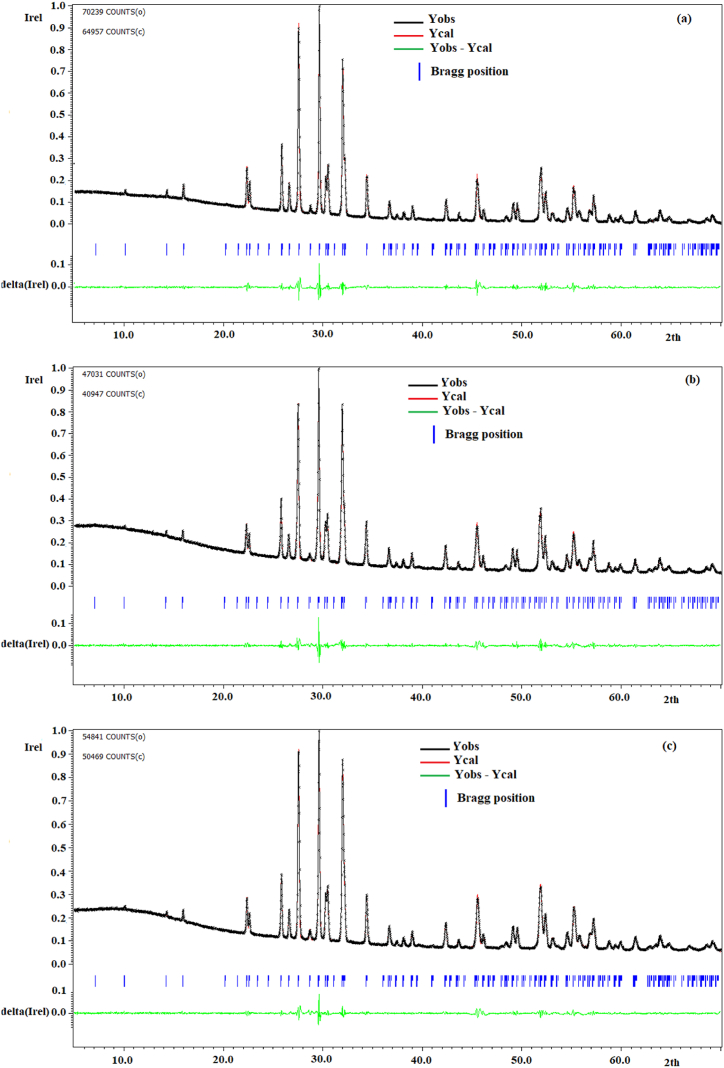
Results of profile refinements for powders synthesized at 1200 °C (a) BNN, (b) BNYN0.02 and (c) BNYN0.04.

For more details on the different structural parameters of the synthesized products, the XRD spectra of the analyzed powders were refined with the Jana 2006 software [38]. The structural model related to the orthorhombic phase of Ba_2_NaNb_5_O_15_ is the starting model used to refine the composition of BNN, BNYN0.02 and BNYN0.04, such as space group (N°. 35), lattice parameters a = 17.58881 Å, b = 17.62308 Å and c = 3.992668 Å [23]. All samples show a pure phase, which can be well indexed to an orthorhombic tungsten bronze structure with a space group *Cmm*2. Fig. 3(a–c) shows the profile refinement of BNN, BNYN0.02 and BNYN0.04 compounds respectively. Table 1 shows the cell parameters (a, b, c, and V) and reliability parameters (Rp (%), Rwp (%), and GOF). The tetragonal-orthorhombic phase transition is accompanied by a 45° rotation about the c-axis of the crystallographic axes, and the crystallographic axes are therefore classified as c < a < b [9]. The reliability factors R_p_ and R_wp_ indicate that the experimental and calculated data are in good agreement and confirm the structural model.

**Table 1:** The structural parameters (a, b, c, V and space group) and the reliability factors of Ba_2_Na_(1-3x)_Y_x_Nb_5_O_15_ ceramics.

Chemical formula	Ba_2_NaNb_5_O_15_	Ba_2_Na_0.94_Y_0.02_Nb_5_O_15_	Ba_2_Na_0.88_Y_0.04_Nb_5_O_15_
a (Å)	17.604(2)	17.596(4)	17.6069(11)
b (Å)	17.624(2)	17.599(5)	17.6299(13)
c (Å)	3.9895(5)	3.9831(3)	3.98450(17)
V (Å^3^)	1237.7(3)	1233.5(5)	1236.82(13)
Temperature	25 °C	25 °C	25 °C
Space group	*Cmm*2	*Cmm*2	*Cmm*2
Symmetry	orthorhombic	orthorhombic	orthorhombic
R_p_ (%)	2.30	1.82	1.67
R_wp_ (%)	3.61	2.88	2.54
GOF	3.11	2.38	2.18

### 3.2 Microstructure of ceramic samples

**Fig. 4:**
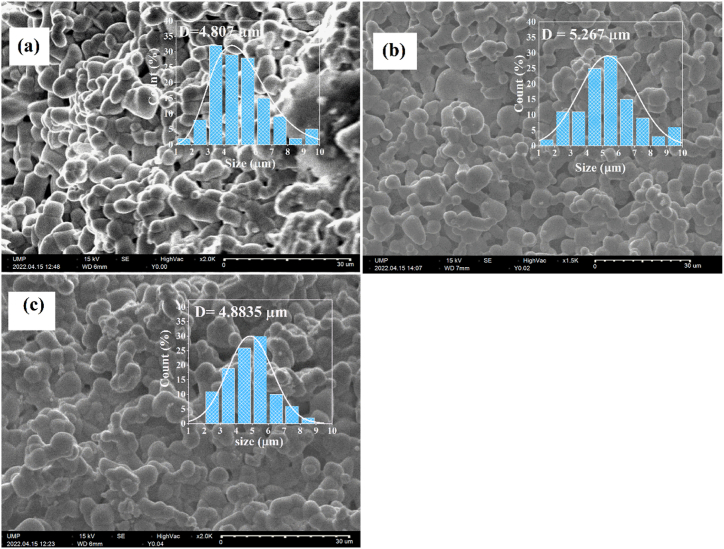
Microstructures of pure and doped Ba_2_NaNb_5_O_15_ ceramics with different concentrations of Yttrium sintered at 1300 °C; (a) BNN, (b) BNYN0.02, and (c) BNYN0.04.

Fig. 4(a–c) shows the micrographs of BNN, BNYN0.02 and BNYN0.04 ceramics, respectively. It is evident in the SEM images that all samples sintered at 1300 °C have a uniform, regulated grain shape without porosities, high densities and well-defined grain boundaries. ImageJ software is used to estimate the average grain size (D). The grain size distribution plots of the samples are shown in the insets in Fig. 3. The average grain size (D) values for BNN, BNYN0.02, and BNYN0.04 are 4.807 μm, 5.267 μm, and 4.8835 μm respectively. The analysis of these values shows that the average grain size increases when Y^3+^ ions partially replace Na^+^ for BNYN0.02 and then decreases for BNYN0.04. In tungsten bronze structure ceramics, a typical feature in morphology is the anisometric pillar-type grain due to the abnormal growth along c axis [11,13,39]. The images we examined show few pillar-type grains, indicating that adding Y^3+^ inhibits abnormal grain growth when a significant amount of Y^3+^ segregates at grain boundaries. The effect in restraining grain growth of Y_2_O_3_ is consistent with the literature [[40], [41], [42]].

### 3.3 Study of dielectric properties

**Fig. 5:**
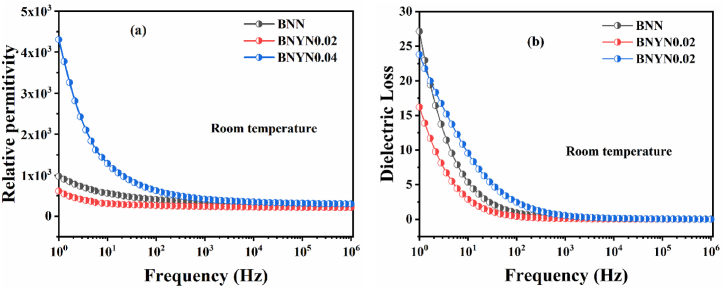
Relative permittivity and dielectric loss as a function of frequency for fritted ceramic samples at 1300 °C: (a) ε′ vs. ƒ; (b) tan(δ) vs. ƒ.

Fig. 5(a–b) shows the evolution of relative permittivity and dielectric loss versus frequency for BNN, BNYN0.02 and BNYN0.04 ceramics at room temperature.

It can be seen that the relative permittivity and dielectric loss first decrease and then increase with increasing Y_2_O_3_ content. When the Y_2_O_3_ content is 0.02, the relative permittivity reaches its minimum value. This phenomenon could be related to the variation of polarization due to the decrease of the cell volume and the increase of the grain size. At low Y_2_O_3_ content, the polarization by ion and electron displacement is weakened, as well as the spontaneous polarization. In addition, the dielectric constant and dielectric loss decrease rapidly with increasing frequency. In general, the highest value of the dielectric constant at low frequency is due to the occurrence of all types of polarizations (ionic, electronic, interfacial, dipolar, etc.) in samples prepared at room temperature [43,44]. Comparison of the relative permittivity and dielectric loss results obtained for our samples with the results obtained in the literature shows similar values to those found for other ceramics as shown in Table 2.

**Table 2:** Comparison of the dielectric properties of BNN, BNYN0.02 and BNYN0.04 with other lead-free ceramics reported in the literature.

Composition	ε′	tan(δ)	Frequency	Reference
Ba_2_NaNb_5_O_15_	98.55	… …	1 KHz	[13]
Ba_1.7_Ba_0.3_NaNb_5_O_15_	531.55	… …	1 KHz	[13]
(Ca_0.28_Ba_0.72_)_2.1_Na_0.8_Nb_5_O_15_	315	0.045	10 KHz	[45]
(Sr_0.925_Ca_0.075_)_2.3_Na_0.4_Nb_5_O_15_	1000	0.013	10 KHz	[46]
(Sr_0.925_Ca_0.075_)_2.05_Na_0.9_Nb_5_O_15_	1500	0.085	10 KHz	[46]
Sr_2_NaNb_5_O_15_	1375	0.027	1 KHz	[47]
Sr_2_Ag_0.2_Na_0.8_Nb_5_O_15_	2000	0.012	1 KHz	[47]
Ba_2_NaNb_5_O_15_	266.683	0.041	10 KHz	Present work
Ba_2_Na_0__.94_Y_0.02_Nb_5_O_15_	217.092	0.015	10 KHz	Present work
Ba_2_Na_0__.88_Y_0.04_Nb_5_O_15_	317.652	0.047	10 KHz	Present work

**Fig. 6:**
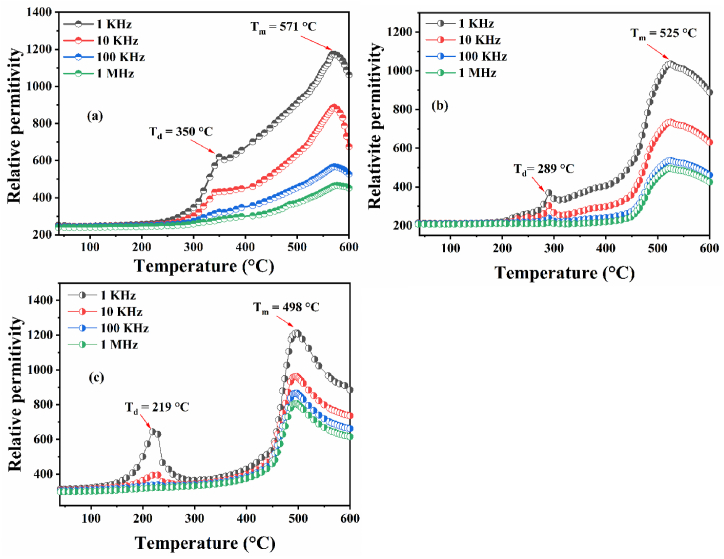
Temperature dependence of relative permittivity for (a) BNN, (b) BNYN0.02, and (c) BNYN0.04.

The dielectric constant ($\varepsilon^{\prime }$) as a function of temperature for Ba_2_Na_(1-3x)_Y_x_Nb_5_O_15_ with (x = 0, 0.02, and 0.04) at different frequencies (1 KHz-1 MHz) in the temperature range between 40 and 600 °C is shown in Fig. 6(a–c).

**Fig. 7:**
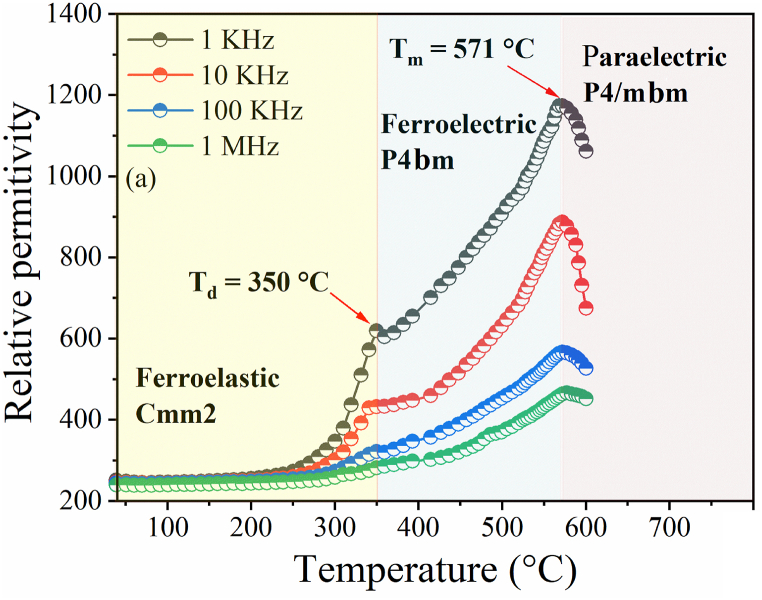
Temperature dependence of relative permittivity for BNN ceramic.

We can see in Fig. 7 corresponds to the variation of the relative permittivity as a function of the temperature of the compound BNN, at about T_m_ = 571 °C the presence of a large peak which is a sign of phase transition from tetragonal ferroelectric (*P*4*bm*) to tetragonal paraelectric (*P*4/*mbm*). Moreover, at about T_d_ = 350 °C, another anomaly appears. This anomaly, which corresponds to the formation of a structural transition from orthorhombic (*Cmm*2) to tetragonal (*P*4*bm*), has been attributed to the ferroelastic-ferroelectric transition [27].

The same anomalies were observed for the BNYN0.02 and BNYN0.04 compounds, but with decreased transition temperatures T_m_ and T_d_ Fig. 6(b) and (c). These results are in good accord with structural studies performed for compounds BNN, BNYN0.02 and BNYN0.04 that show an orthorhombic structure of the *Cmm*2 space group at room temperature.

**Fig. 8:**
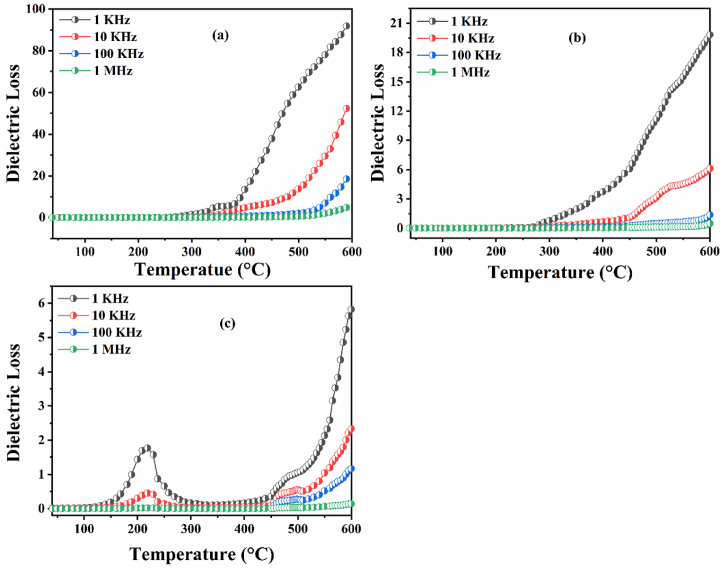
The evolution of the dielectric loss as a function of temperature: (a) BNN, (b) BNYN0.02, (c) BNYN0.04.

Fig. 8(a–c) illustrates the variation of the dielectric loss with temperature, for all the samples studied. In Fig. 8(a–c), In low temperature regions, it is obvious that the dielectric loss increases slowly with temperature and then increases significantly at high temperatures. This behavior may be due to the improved charge transport characteristics in the prepared ceramics that are thermally activated during the temperature increase, i.e. the increase of the charge carriers mobility by the thermal activation [48].

In other words, the observed characteristics have a solid practical relationship with the phase transition observed for the prepared composition; precisely for the compound BNYN0.04 it is very clear that this sample undergoes two anomalies in the temperature range 40 °C–600 °C related to the phase transitions indicated previously in the part of the dielectric constant, we also notice the presence of the phenomenon of dielectric relaxation associated with the migration of charge carriers. At high temperatures and low frequencies, the observed dielectric loss increase can be attributed to the polarization of space charges [49]. The highest values of the dielectric parameters (ε' & tanδ) observed above 300 °C, may be related to the formation of oxygen vacancies in the prepared ceramics. The oxygen vacancies play an important role in changing the values of relative permittivity and dielectric loss, which may control the properties of electric charge carriers present in these materials. The substitution of Na^+^ ions by Y^3+^ ions in the square sites of the BNN structure leads to the creation of ionized vacancies of sodium ($V_{\text{Na}}^{\prime \prime }$) similarly to the substitution of bivalent ions such as Ba^2+^,Ca^2+^,Sr^2+^, … by trivalent ions such as rare earth (La^3+^,Sm^3+^, …) [[50], [51], [52]].

equation (1) that shows the formation of the sodium vacancies ($V_{Na}^{\prime \prime }$) is the following:(1)
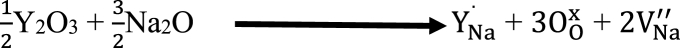


### 3.4 Complex impedance analysis (CIA)

#### 3.4.1 Nyquist plot

Complex impedance analysis, using Nyquist diagrams to understand grain properties, grain boundaries and possible electrode effects on resistive, capacitive, inductive and reactive material properties [53]. The complex impedance Z* can be obtained from the expression (2) below:

(2) Z* = Z^'^+ jZ^''^

Where, Z′ and Z″ represent respectively the real and the imaginary part of the impedance and $j=\sqrt{-1}$ is the imaginary factor [19]. The expressions (3, 4) of Z′ and Z″ are respectively the following:

(3) $$Z^{\prime }=\frac{R_{e}}{1+\omega^{2}+C_{e}^{2}+R_{e}^{2}}+\frac{R_{\text{gb}}}{1+\omega^{2}+C_{\text{gb}}^{2}+R_{\text{gb}}^{2}}+\frac{R_{g}}{1+\omega^{2}+C_{g}^{2}+R_{g}^{2}}$$

(4) $$Z^{\prime \prime }=\left[\frac{\omega {C_{e}R}_{e}}{1+\omega^{2}+C_{e}^{2}+R_{e}^{2}}+\frac{\omega {C_{\text{gb}}R}_{\text{gb}}}{1+\omega^{2}+C_{\text{gb}}^{2}+R_{\text{gb}}^{2}}+\frac{\omega {C_{g}R}_{g}}{1+\omega^{2}+C_{g}^{2}+R_{g}^{2}}\right]$$

**Fig. 9:**
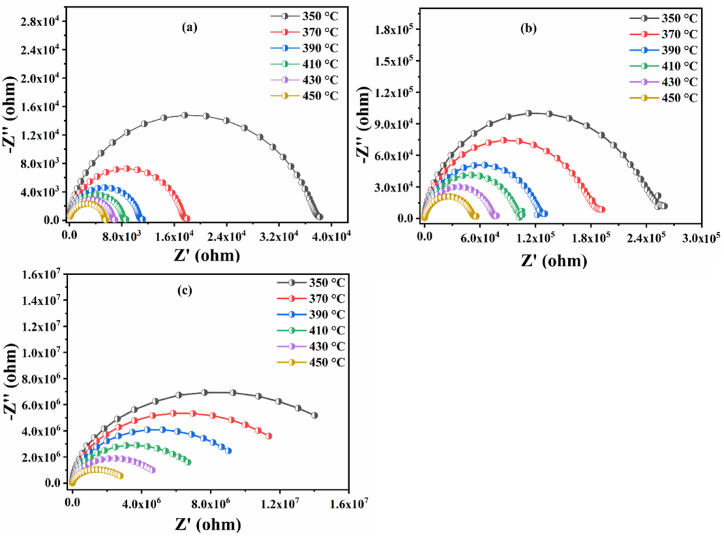
Complex impedance diagram at different temperatures for different Y contents in Ba_2_Na_(1-3x)_Nb_5_O_15_; (a) BNN, (b) BNYN0.02 and (c) BNYN0.04.

Where, R_(g, gb, and e)_ represent the resulting resistance of grains, grain boundaries, and electrode effects, respectively, while C_(g, gb, and e)_ represents the resulting capacitance of the same components [19]. Fig. 9(a–c) shows Nyquist plots (plots of the imaginary part against the real part of the impedance) for BNN, BNYN0.02, and BNYN0.04 ceramics at different temperatures.

The Nyquist plots (Cole-Cole) show well resolved semicircles for all temperatures studied [350 °C–450 °C]. As the temperature increases, one can observe that the rayon of the semicircles decreases, indicating a decrease in Resistance (R). This behavior confirms that the conduction process is thermally activated in these materials, and also the semiconducting nature of our samples [12,19,53,54].

**Fig. 10:**
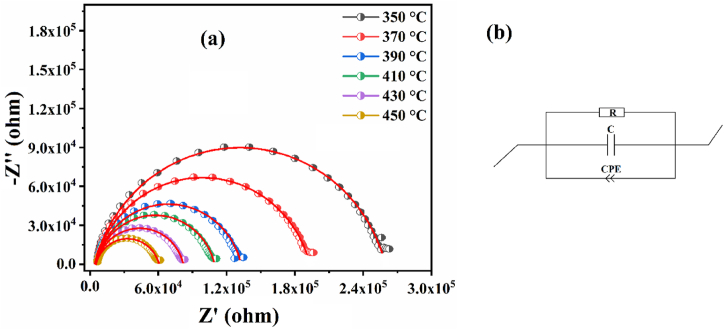
(a) Plot of the imaginary part (Z″) versus the real part (Z′) fitted with the MT-Lab software for the composition of BNYN0.02 at different temperatures. (b) Represents the equivalent electrical circuits corresponding to the response of the grains.

The experimental Nyquist diagrams obtained are poorly fitted by an electrical model in order to find the values of various electrical parameters of the proposed electrical circuit. The model of the equivalent circuit that we suggests related to the contribution of grains composed of Capacitors (C_g_), Resistors (R_g_) and Constant Phase Element (CPE_g_) is shown in Fig. 10(b).

However, the non-ideal capacitive behavior of our samples is confirmed by the presence of a CPE in the equivalent circuit (non-Debye type relaxation) [53,55]. The correlation between CPE and C is expressed by the following equation (5):

(5) $$C=R^{\left(\frac{1-\alpha }{\alpha }\right)}\times {\text{CPE}}^{\frac{1}{\alpha }}$$

Where α is the degree of energy dissipation (α < 1), the value of α is between 0 and 1 for non-ideal capacitive behavior, α = 1 for a pure capacitor and α = 0 for a pure resistor [56]. The relation that allows us to calculate the depression angle β caused by the CPE phase element is the following: β = $\frac{\pi }{2}$ (1-α) [57]. Fig. 10(a) which shows the impedance spectrum fitted with the MT-Lab software of the BNYN0.02 ceramic at different temperatures. The results obtained at different temperatures for the prepared compounds are presented in Table 3.

**Table 3:** Nyquist plot fitting results for BNN, BNYN0.02 and BNYN0.04 ceramics at different temperatures.

Compound	T(°C)	R (Ω)	CPE (F.s^(a-1)^)	C	α	β (°)
BNN	350	38390	10.89 10^−9^	0.2015 10^−9^	0.6289	33.399
	360	25515	10.12 10^−9^	0.2038 10^−9^	0.6368	32.688
	370	17813	8.584 10^−9^	0.2048 10^−9^	0.6528	31.248
	380	13385	8.272 10^−9^	0.2066 10^−9^	0.6584	30.744
	390	11000	7.657 10^−9^	0.2078 10^−9^	06680	29.880
	400	9560	8.358 10^−9^	0.2100 10^−9^	0.6641	30.231
	410	8474	7.440 10^−9^	0.2103 10^−9^	0.6768	29.088
	420	7630	6.642 10^−9^	0.2092 10^−9^	0.6914	27.774
	430	6824	5.432 10^−9^	0.2054 10^−9^	0.7151	25.641
	440	6167	4.194 10^−9^	0.1977 10^−9^	0.7443	23.013
	450	5509	3.372 10^−9^	0.1871 10^−9^	0.7718	20.538
BNYN0.02	350	258918	5.244 10^−9^	0.1959 10^−9^	0.6301	33.291
	360	225921	5.138 10^−9^	0.1953 10^−9^	0.6384	32.544
	370	190143	5.262 10^−9^	0.1950 10^−9^	0.6427	32.157
	380	150752	5.447 10^−9^	0.1948 10^−9^	0.6442	32.022
	390	126792	5.493 10^−9^	0.1957 10^−9^	0.6450	31.950
	400	120931	6.091 10^−9^	0.1981 10^−9^	0.6377	32.607
	410	104989	6.848 10^−9^	0.2008 10^−9^	0.6302	33.282
	420	90271	7.289 10^−9^	0.2028 10^−9^	0.6303	33.273
	430	76937	9.006 10^−9^	0.2092 10^−9^	0.6168	34.488
	440	64250	11.270 10^−9^	0.2130 10^−9^	0.6065	35.415
	450	55 083	13.760 10^−9^	0.2170 10^−9^	0.5988	36.108
BNYN0.04	350	17.36 10^6^	0.862 10^−9^	0.1997 10^−9^	0.5967	36.108
	360	15.74 10^6^	1.553 10^−9^	0.2036 10^−9^	0.5330	36.297
	370	13,70 10^6^	1.319 10^−9^	0.2056 10^−9^	0.5635	42.030
	380	12.37 10^6^	1.408 10^−9^	0.2084 10^−9^	0.5687	39285
	390	10.8 10^6^	1.708 10^−9^	0.2124 10^−9^	0.5563	38.817
	400	9.53 10^6^	1.767 10^−9^	0.2163 10^−9^	0.5684	39.933
	410	7.91 10^6^	2.397 10^−9^	0.2229 10^−9^	0.5476	38.844
	420	6.63 10^6^	4.088 10^−9^	0.2311 10^−9^	0.5064	40.716
	430	5.45 10^6^	4.010 10^−9^	0.2381 10^−9^	0.5298	44.424
	440	4.29 10^6^	5.696 10^−9^	0.2495 10^−9^	0.5132	42.318
	450	3.40 10^6^	9.263 10^−9^	0.2656 10^−9^	0.5176	43.412

**Fig. 11:**
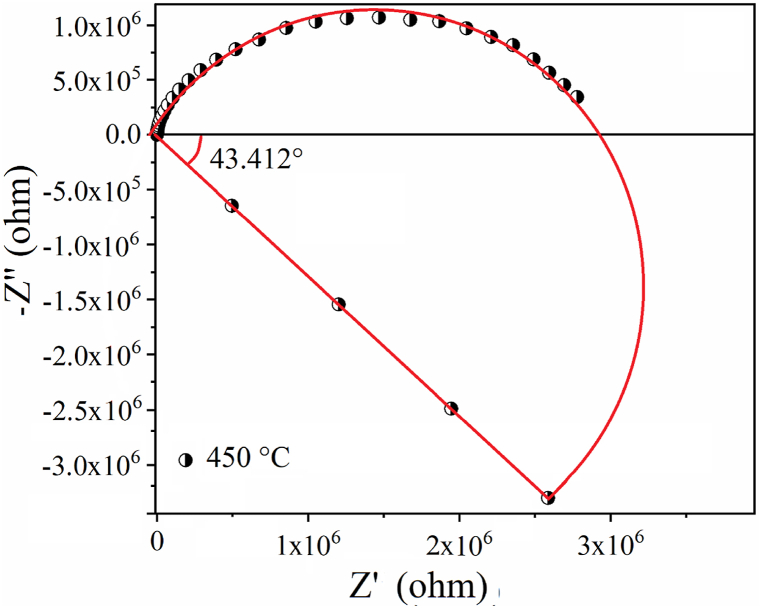
Verification of experimental results of Nyquist traces for BNYN0.04 ceramic at 450 °C as a function of depression angle β.

Furthermore, the representation of a semicircle for the compound BNYN0.04 at 450 °C (see Fig. 11) allows confirmation of the deviation from the Debye behavior, it was established that the sample deviates from the Debye behavior by an angle of 43.412°.

In general, Nyquist traces (-Z″ vs. Z′) include multiple semicircles in different frequency regions, i.e., three semicircles represent the existence of an electrode effect, a grain boundary effect, and a bulk (grain) effect, two semicircles represent the existence of a grain boundary effect and a grain effect, and one semicircle represents the existence of a grain effect [58,59]. To study the charge carrier activity at the grain and grain boundary level, the activation energies of the ceramics can be calculated using the following equation (6) [[60], [61], [62]]:

(6) R=R_0_exp(E_1,2_/k_B_T)

**Fig. 12:**
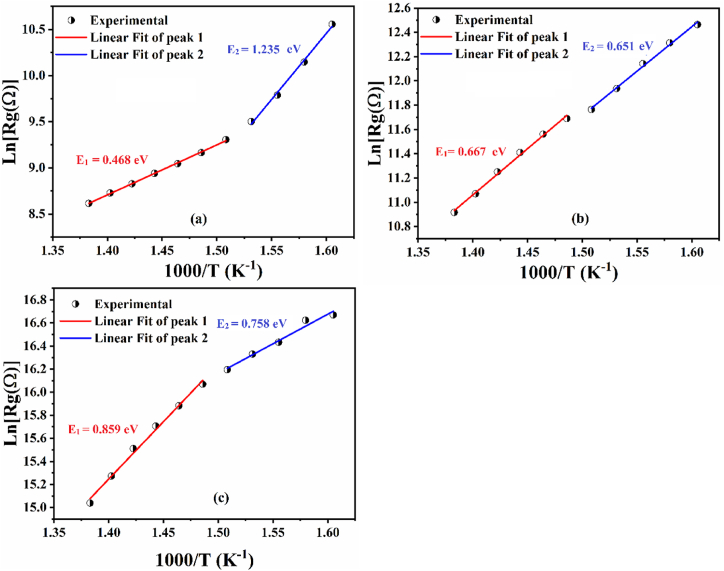
Arrhenius plots of Ba_2_Na_(1-3x)_Y_x_Nb_5_O_15_ ceramics as a function of 1000/T in the temperature range [ 350 °C–450 °C]: (a) BNN; (b) BNYN0.02 and (c) BNYN0.04.

Where, R is the resistance of the grains of the material obtained by the complex impedance, R_0_ is the pre-exponential constant, k_B_ is the Boltzmann constant and T is the test temperature. The activation energies are determined from the slope of the lines obtained by plotting Ln (R_g_) versus 1000/T as shown in Fig. 12(a–c).

Two linear slopes are observed in Fig. 12, i.e., two different temperature regions, which can be explained by the contribution of two conduction processes to the electrical properties of BNN ceramics, BNYN0.02 and BNYN0.04. The E_1_ and E_2_ values obtained by linear fitting for BNN, BNYN0.02 and BNYN0.04 ceramics are 0.468, 0.66, 0.859 eV, and 1.235, 0.651, 0.758 eV respectively.

The activation energies corresponding to region 1 and region 2 are similar to those obtained by electrical conductivity. The origin of these two activation energies will be detailed in the discussion of electrical conductivity.

#### 3.4.2 The real and the imaginary part of the impedance

**Fig. 13:**
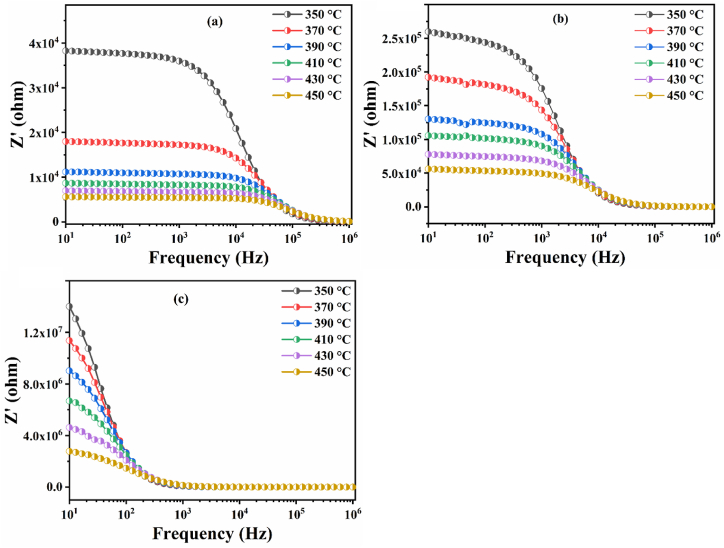
The traces of the evolution of (Z′) as a function of frequency at different temperatures for Ba_2_Na_(1-3x)_Y_x_Nb_5_O_15_ ceramics: (a) BNN; (b) BNYN0.02 and (c) BNYN0.04.

The variation of the real part of the impedance (Z′) with frequency at different temperatures is shown in Fig. 13(a–c). It can be observed that the value of the Z′ decreases with increasing temperature and frequency, suggesting an increase in electrical conductivity as well as a decrease in electrical resistance. This type of behavior confirms the presence of the negative temperature coefficient of resistivity (NTCR) in all prepared materials [12,63].

On the other hand, for all temperatures and in the high frequency region, the Z′ values merge and then become frequency independent, this can be due to the release of a space charge due to the reduction of the barrier properties of the material [63]. The remarkable decrease in barrier properties in our materials with increasing temperature can be justified by the increased AC conductivity of the materials at high frequencies [64,65]. This particular frequency at which the value of Z′ becomes frequency independent and shifts to the low frequency side is caused by the insertion of Y^3+^ into the base compound BNN; this indicates that the Y^3+^ doped compounds begin to release space charge from the lowest frequencies: 10^4^ Hz for BNYN0.02 and less than 10^3^ Hz for BNYN0.04.

**Fig. 14:**
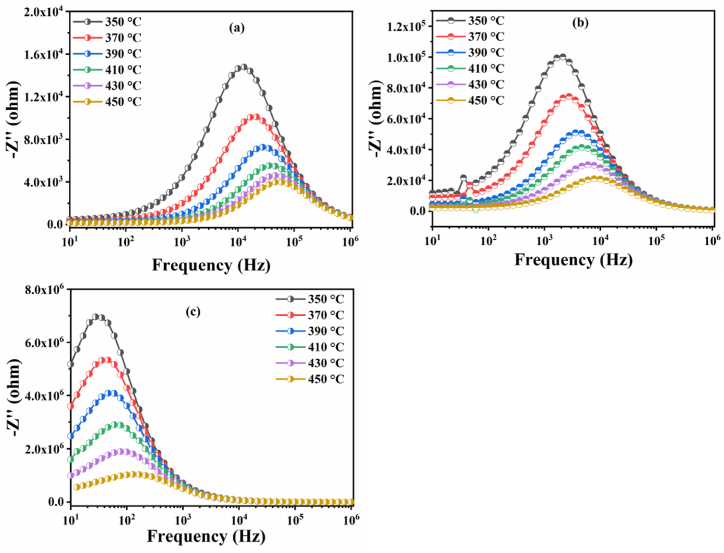
Curves of the imaginary part of the impedance (-Zʺ vs ƒ) at different temperatures (350 °C–450 °C) for (a) BNN, (b) BNYN0.02 and (c) BNYN0.04.

Fig. 14(a–c) shows the variation of the imaginary part of the impedance (Z″) as a function of frequency at different temperatures (350 °C–450 °C) for BNN, BNYN0.02 and BNYN0.04 ceramics.

The plots of (-Z″) as a function of frequency (1Hz-1MHz) show two aspects: (I) the relaxation frequency related to the maximum peak of each temperature studied. The presence of a relaxation mechanism in the prepared ceramics is confirmed by the broadening of the corresponding peak at each temperature. The leading cause of the creation of this mechanism is the thermal activation of charge carriers at high temperature, (II) the intensity of the -Z″ peak as a function of frequency decreases with increasing temperature and shifts to higher frequency values, suggesting that the relaxation process is thermally activated with space charge accumulation at the barrier [[66], [67], [68], [69], [70]]. The relaxation frequency ƒ_max_ and the relaxation time τ are related to each other by the following relationship τ = $\frac{1}{2\pi .{ƒ}_{\max}}$. Therefore, the relaxation frequency ƒ_max_ and the relaxation time τ are inversely proportional. Seeing that the highest ƒ_max_ frequency is that of the BNN compound, it is therefore very easy to confirm that the longest relaxation time τ is that of the BNYN0.04 sample. In addition, the mobility of oxygen vacancies is reduced as the relaxation time increases [71]. which indicates that the mobility of the oxygen vacancies is weakened by the substitution of the sodium ion Na^+^ by the yttrium ion Y^3+^ and this can be confirmed by the intensity of the relaxation peak which is proportional to the concentration of the mobile oxygen vacancies [72].

### 3.5 Electrical resistivity

One of the most important parameters to consider in high-temperature applications is electrical resistivity. Electrical resistivity ρ was calculated from material resistance data and the geometric parameters of the samples [73]. Equation (7) is used to calculate ρ.

(7) $$\rho =R_{\text{material}}\frac{A}{t}$$

**Fig. 15:**
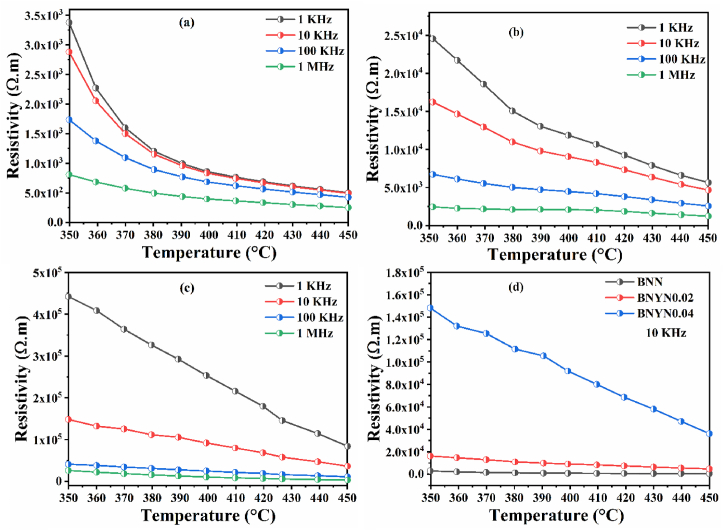
Temperature dependence of resistivity for ceramics prepared between 350 °C and 450 °C, (a) BNN, (b) BNYN0.02, (c) BNYN0.04 and (d) comparison of resistivity at 10 KHz.

Where $R_{\text{material}}$ is the material resistance, t the sample thickness and A the electrode surface. Fig. 15(a, b and c) shows the variation of electrical resistivity ρ as a function of temperature for BNN, BNYN0.02 and BNYN0.04. As can be seen, the resistivity of the prepared ceramics decreases with increasing temperature where the value of ρ at 1 KHz decreases from 3.38 × 10^3^ Ω m (350 °C) to 5.02 × 10^2^ Ω m (450 °C) for BNN, from 2.45 × 10^4^ Ω m (350 °C) to 5.65 × 10^3^ Ω m (450 °C) for BNYN0.02 and from 4.50 × 10^5^ Ω m (350 °C) to 8.41 × 10^4^ Ω m (450 °C) for BNYN0.04. This shows that resistivity depends on the Negative Temperature Coefficient Response (NTCR) and also indicates semiconductor-like behavior for all prepared samples [74,75].

Fig. 15(d) shows the effect of substituting sodium by yttrium on the resistivity of different compositions at different temperatures. Resistivity increases significantly with increasing Y^3+^ content. BNYN0.04 ceramics have the highest resistivity (1.5 × 10^5^ Ω m 350 °C and 3.6 × 10^4^ Ω m 450 °C), which is superior to that of pure BNN ceramics at 10 KHz. The increase in electrical resistivity observed for compounds containing Y^3+^ ions is explained by the decrease in the concentration of oxygen vacancies (OVs) in these compounds. Oxygen vacancies can be doubly ionized, singly ionized or neutral. They are mobile charge carriers that play an important role in the conduction process [76,77]. The improvement in resistivity is accompanied by a decrease in electrical conductivity. a similar behavior was observed for perovskite structure oxides containing titanium ions in which DC conductivity decreases with the reduction of oxygen vacancies [78,79].

### 3.6 Electrical conductivity analysis

The conductivity AC is one of the factors that control the conduction mechanisms in this type of material and to better understand this mechanism, (σ_AC_) is therefore determined by the following equation (8) [61]:

(8) $$\sigma_{\text{AC}}=\omega \varepsilon_{0}\varepsilon^{\prime \prime }$$

**Fig. 16:**
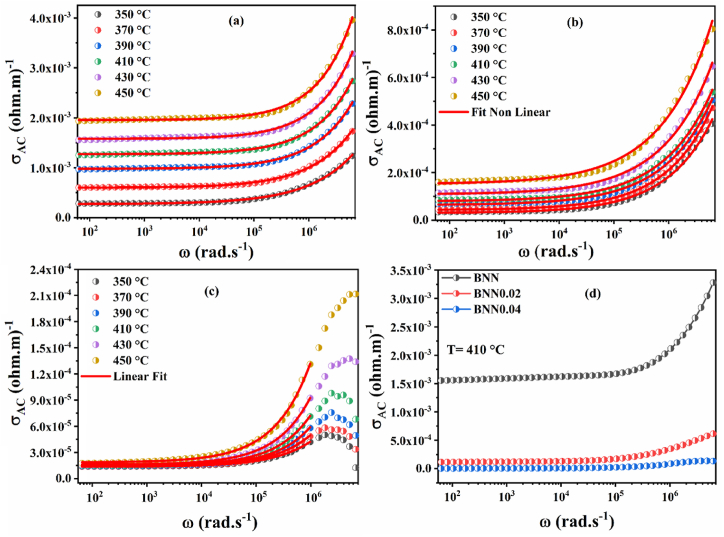
Conductivity AC as a function of frequency at different temperatures (350 °C–450 °C) for (a)BNN, (b)BNYN0.02, (c)BNYN0.04 and (d) comparison of conductivity AC at T = 410 °C for BNN, BNYN0.02 and BNYN0.04. The red lines show the fit by Jonscher's power law. (For interpretation of the references to color in this figure legend, the reader is referred to the Web version of this article.)

Where, ω is the angular frequency, ε'' is the imaginary part of the material permittivity and $\varepsilon_{0}$ is the vacuum permittivity. Fig. 16(a–c) includes the ac conductivity analysis of undoped and Y_2_O_3_-doped BNN ceramics in the temperature range of 350 °C–450 °C.

We can observe in Fig. 16(a–c) two different regions, the first region is related to low frequencies that form a plateau, while the second region at high frequencies that is characterized by a gradual increase in conductivity, and on the other hand, we see no dispersion of conductivity. Therefore, we can conclude that the short-range movement of ionic species has replaced the long-range jump in the samples [12].

To fully understand the conduction mechanisms and the species responsible for conduction in our samples, we fitted the AC conductivity by Jonscher's power law using equation (9) [9,[80], [81], [82]].

(9) $$\sigma_{ac}=\sigma_{\text{dc}}+A\omega^{n}$$

**Fig. 17:**
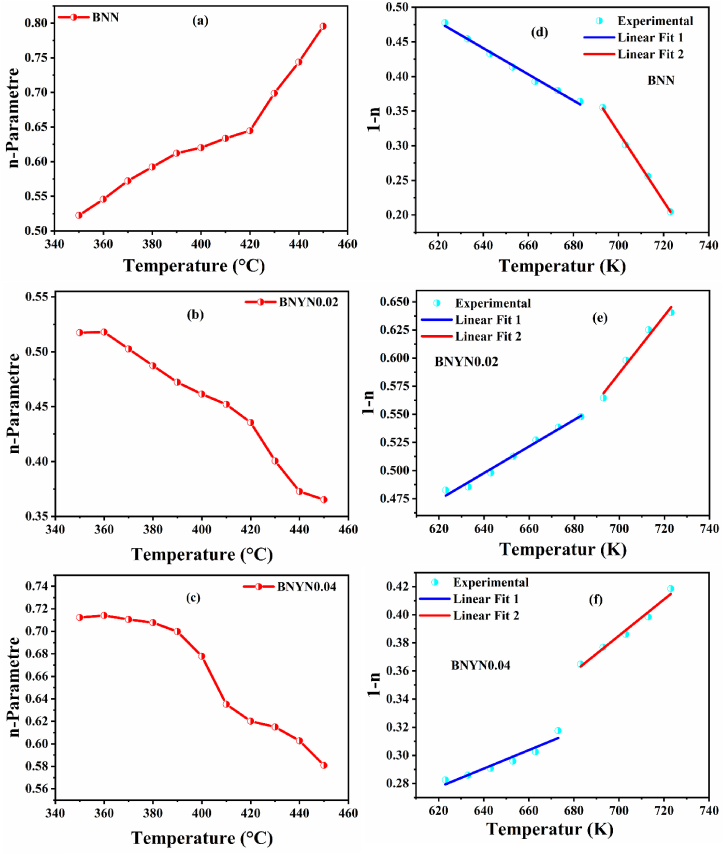
The variation of the parameters ''n'' and ''1-n'' as a function of temperature: (a and d) BNN, (b and e) BNYN0.02 and (c and f)) BNYN0.04.

Where σ_dc_ is the conductivity in the continuous current of the material, ''A'' is the pre-exponential factor that determines the polarization strength and the parameter ''n'' which is independent of the frequency and depends on two factors: temperature and intrinsic material property [83]. The determination of the value of "n" is very important to understand the different conduction mechanisms in our materials and the transport properties of charge carriers (vacancies, electrons, ions …). If ''n'' is superior to 1 (n > 1), it means that the charge carriers move with a localized jump without the species leaving the vicinity, if n is inferior to 1 (n < 1), it means that the charge carriers move with a translational motion (a sudden jump) [[84], [85], [86]]. The red lines in Fig. 17(a–c) show the nonlinear curves fitted to Equation (9) of the Jonscher power law for compositions BNN, BNYN0.02, and BNYN0.04, respectively. The values of the fitting parameters A, n, and σ_dc_ for all compositions in the temperature range of 350 °C–450 °C are shown in Table 4.

**Table 4:** The parameters ($\sigma_{\text{dc}},A$ andn) fitted by the Jonscher power law at different temperatures (350 °C–450 °C) for BNN, BNYN0.02 and BNYN0.04 ceramics.

Composition	T(°C)	$\sigma_{\text{dc}}$ (Ω.m)^−1^	A (Ω^−1^ m^−1^ rad^-s^)	n
BNN	350	2.7237610^−4^ ± 2.37231 10^−6^	2.77173 10^−7^± 2.83516 10^−8^	0.52249
	360	4.1531810^−4^ ± 2.41789 10^−6^	2.08725 10^−7^± 2.15679 10^−8^	0.54573
	370	5.9927810^−4^ ± 2.56256 10^−6^	1.49156 10^−7^ ± 1.63069 10^−8^	0.57223
	380	8.0062810^−4^ ± 2.75155 10^−6^	1.17292 10^−7^± 1.34974 10^−8^	0.59244
	390	9.7638110^−4^ ± 2.91142 10^−6^	9.20262 10^−8^± 1.10491 10^−8^	0.61220
	400	1.12 10^−3^ ± 3.1988 10^−6^	8.63121 10^−8^± 1.09253 10^−8^	0.62028
	410	1.27 10^−3^ ± 3.54192 10^−6^	7.375 10^−8^ ± 1.01429 10^−8^	0.63372
	420	1.41 10^−3^ ± 3.90179 10^−6^	6.62963 10^−8^± 9.65189 10^−9^	0.64484
	430	1.58 10^−3^ ± 3.51647 10^−6^	3.25983 10^−8^± 5.70092 10^−9^	0.69885
	440	1.75 10^−3^ ± 3.36119 10^−6^	1.81983 10^−8^ ± 3.55931 10^−9^	0.74393
	450	1.96 10^−3^ ± 3.09737 10^−6^	9.38951 10^−9^ ± 2.00335 10^−9^	0.79559
BNYN0.02	350	3.03664 10^−5^ ± 1.30233 10^−6^	1.19831 10^−7^ ± 1.65808 10^−8^	0.51742
	360	3.48891 10^−5^ ± 1.41773 10^−6^	1.28294 10^−7^ ± 1.79356 10^−8^	0.51793
	370	4.10836 10^−5^ ± 1.80174 10^−6^	1.6976 10^−7^± 2.75535 10^−8^	0.50266
	380	5.23805 10^−5^ ± 2.06061 10^−6^	2.18396 10^−7^ ± 3.81211 10^−8^	0.48719
	390	6.1697 10^−5^ ± 2.3391 10^−6^	2.71077 10^−7^ ± 5.19183 10^−8^	0.47224
	400	6.81916 10^−5^ ± 2.4278 10^−6^	3.90658 10^−7^ ± 6.13901 10^−8^	0.46145
	410	7.60097 10^−5^ ± 2.69629 10^−6^	3.78001 10^−7^ ± 7.62651 10^−8^	0.45210
	420	8.95572 10^−5^ ± 2.78751 10^−6^	3.74515 10^−7^ ± 7.57156 10^−8^	0.43548
	430	1.07384 10^−4^ ± 2.49975 10^−6^	3.4053 10^−7^ ± 5.66497 10^−8^	0.40053
	440	1.29679 10^−4^ ± 2.75685 10^−6^	3.22131 10^−7^ ± 6.0849 10^−8^	0.37270
	450	1.5224210^−4^ ± 2.98055 10^−6^	4.03187 10^−7^ ± 6.37688 10^−8^	0.36527
BNYN0.04	350	1.0038 10^−6^ ± 3.68779 10^−8^	1.23868 10^−9^ ± 1.06136 10^−10^	0.71234
	360	1.1407 10^−6^ ± 5.25553 10^−8^	2.6071 10^−9^ ± 3.49243 10^−10^	0.71409
	370	1.37391 10^−6^ ± 7.2504 10^−8^	2.84658 10^−9^ ± 3.59608 10^−10^	0.71051
	380	1.57401 10^−6^± 7.70191 10^−8^	3.76103 10^−9^ ± 2.99941 10^−10^	0.70765
	390	1.66877 10^−6^ ± 1.02774 10^−7^	4.15162 10^−9^ ± 4.45048 10^−10^	0.69980
	400	2.02439 10^−6^ ± 1.24057 10^−7^	5.85097 10^−9^ ± 4.52671 10^−10^	0.67786
	410	2.18672 10^−6^± 2.67079 10^−7^	9.68469 10^−9^ ± 1.63816 10^−9^	0.63517
	420	2.37753 10^−6^ ± 3.70202 10^−7^	1.33809 10^−8^ ± 2.70083 10^−9^	0.62022
	430	3.26017 10^−6^ ± 2.80396 10^−7^	1.44413 10^−8^± 1.53307 10^−9^	0.61502
	440	4.44542 10^−6^ ± 2.04112 10^−7^	1.62718 10^−8^ ± 1.95942 10^−9^	0.60274
	450	6.59789 10^−6^ ± 3.95017 10^−7^	1.88092 10^−8^ ± 2.0709 10^−9^	0.58087

The traces of n as a function of temperature n(T) can be used to determine the conduction pattern in the prepared samples. In the literature, many researchers have reported several types of models [85,86]. Fig. 17(a–c) represents the temperature dependence of the ''n'' parameter (350 °C–450 °C) and Fig. 17(d–e) shows the temperature variation of ''1-n'' for undoped and Y_2_O_3_-doped BNN compounds.

In Fig. 17 (a), it can be seen that the exponent n increases with increasing temperature, indicating that Non-overlapping Small Polaron Tunneling (NSPT) is the suitable model to describe the mechanism of electrical conduction (charge transport) in the base compound BNN. According to this model, the increase of the frequency leads to the decrease of the tunneling distance until reaching the minimum value which is still non-zero, but equivalent to the dispersion between the atoms. And also, the conductivity AC in this sample is due to the addition of a charge carrier on a site that causes a local distortion of the lattice [85]. The W_h_ energy of the polaron jumps from one position to another related to the NSPT model was established from the linear fit of the (1-n_NSPT_) experimental data Fig. 17(d) with $n_{\text{NSPT}}$ = 1+ $\frac{4\times K_{B}\times T}{W_{h}}$ [87,88]. It was very clear that Fig. 17(d) shows two different regions which suggest that two jump energies of the polarons $W_{h}^{1}$ and $W_{h}^{2}$ exist, this can be explained by the presence of two types of polarons in the BNN material. The values found for $W_{h}^{1}$ and $W_{h}^{2}$ are respectively $W_{h}^{1}$ = 0.182 eV and $W_{h}^{2}$ = 0.069 eV. Furthermore, in Fig. 17 (b and c), we observe that the exponent ''n'' is inversely proportional to the increase in temperature. This indicates that the conduction model of charge transport in Y_2_O_3_-doped compounds (BNYN0.02 and BNYN0.04) is Correlated with Barrier Hopping (CBH) [89]. According to this model, charge carriers can move from one site to another through thermal activation, crossing the potential barrier between them. This mechanism plays an essential role in the regulation of the AC conductivity observed in these samples [84]. Similarly, we have calculated the barrier height W_m_ for the CBH model Fig. 17(f et e) with n_CBH_ = 1- $\frac{6\times K_{B}\times T}{W_{m}}$ [90]. The values found for $W_{m}^{1}$ and $W_{m}^{2}$ of the compounds BNYN0.02 and BNYN0.04 are $W_{m}^{1}$ = 0.438 eV, $W_{m}^{2}$ = 0.203 eV and $W_{m}^{1}$ = 0.783 eV, $W_{m}^{2}$ = 0.401 eV respectively.

The conductivity dc of the prepared samples is increased with temperature as shown in Table 4. This indicates that this type of conductivity exhibits Arrhenius behavior which can be written according to equation (10) [66]:

(10) $$\sigma_{\text{dc}}=\sigma_{0}\;\mathrm{Exp}\left(\frac{-E_{1,2}}{k_{B}\;T}\right)$$

**Fig. 18:**
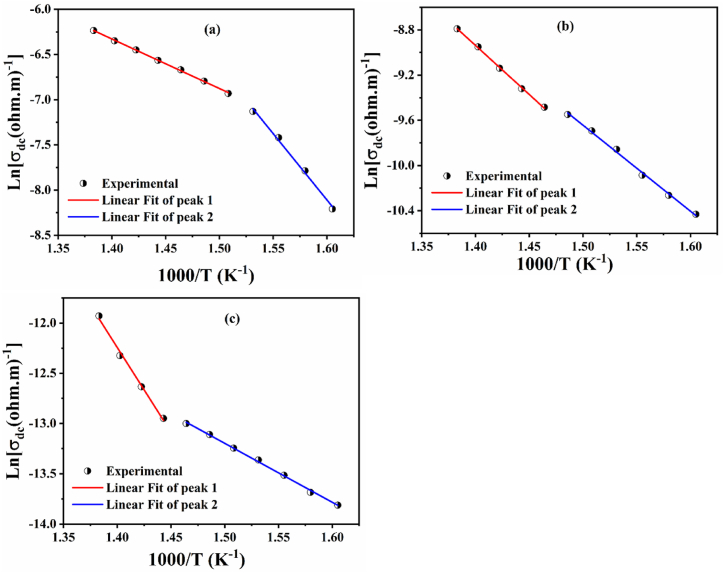
The variation of ln(σ_dc_) as a function of 1000/T for; (a) BNN, (b) BNYN0.02, and (c) BNYN0.04 ceramics.

Where σ_0_ is a pre-exponential factor, k_B_ and $E_{1,2}$ are the Boltzmann constant and the activation energy, respectively. The activation energies obtained by the slope of the linear fit curves (Ln(σ_dc_) vs10^3^/T) are shown in Fig. 18 (a,b and c).

The plots of Ln(σ_dc_) versus 1000/T show two different regimes with two different slopes for the three ceramics, indicating that each compound has two types of activation energies corresponding to the two different types of charge carriers, the activation energy values found E_1_ (region 1) and E_2_ (region 2) are grouped in Table 5.

**Table 5:** The energy of activation by conduction of the elaborated ceramics.

Composition	$E_{1}$ (eV)	$E_{2}$ (eV)
BNN	0.473	1.262
BNYN0.02	0.749	0.656
BNYN0.04	0.982	0.505

We all know that E_dc_ is the sum of charge carrier generation and long-distance charge carrier migration, or jump free energy [77,91]. With increasing Y^3+^ substitution in the A2 site, the Edc values of BaNa_(1-3x)_Y_x_Nb_5_O_15_ ceramics (x = 0.00, 0.02, 0.04) increase progressively in region 1 and decrease in region 2. For ferroelectric ceramics, the activation energy for the interaction of small polarons lies between 0.2 and 1.5 eV [92]. In addition, the activation energy for the conduction of small polarons is influenced by the movement of the domain walls, which in turn depends on the potential barriers present in the grain boundaries that are more dominated at high temperatures. These potential barriers reflect the deformation of the crystal lattice and the grain size of the material, which can have an impact on the mobility of small polarons [93]. Consequently, the increase in activation energy E_1_ for compounds BNYN0.02 and BNYN0.04 is caused by the higher Y^3+^ content in the grain boundaries, which provides more channels for carrier transport, particularly for long-distance migration. In addition, the activation energy E_2_ related to the grain response decreased due to the low charge carrier concentrations resulting from the Y^3+^ deficiency in these regions.

In general, the thermal activation energy is controlled by the oxygen vacancies ($V_{o}^{˙˙}$) in this type of ferroelectric oxide [91]. It is well known that during the sintering process of ferroelectric perovskites, oxygen vacancies can occur due to the release of oxygen from the crystal lattice [94]. equation (11) that shows the formation of the oxygen vacancies ($V_{o}^{˙˙}$) is the following:(11)
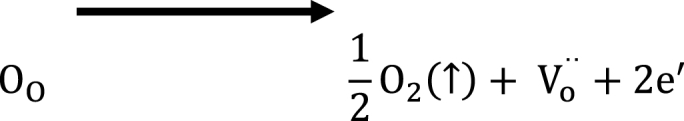


For stoichiometric ABO_3_ perovskites, the activation energy E_a_ is about 2 eV, while the E_a_ value for non-stoichiometric ABO_2.95_ and ABO_2.90_ perovskites is 1 eV and 0.5 eV respectively [80,95]. For the ceramics prepare BNN, BNYN0.02 and BNYN0.04, the activation energy values E_1_ are 0.473, 0.749 and 0.982 eV, respectively, and the activation energy values E_2_ are 1.262, 0.656 and 0.505 eV, which suggests that conduction in our samples is controlled by oxygen vacancies. Moreover, it is well known that the most mobile ionic species in ferroelectric oxides (tungsten bronze, perovskite, spinel) are single (0.3–0.5eV) and doubly ionized (0.6–1.2 eV) oxygen vacancies [80,[96], [97], [98], [99]]. We find that the activation energy values decrease for grains and increase for grain boundaries with increasing Y^3+^ concentration, suggesting that these ions are incorporated into the crystal lattice and that the type of species responsible for the conduction mechanism in ceramics changes with increasing Y^3+^ concentration.

The conducting electrons created by the single and doubly ionized oxygen vacancies are written according to equations (12), (13) [50]:(12)
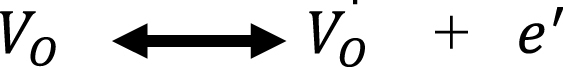
(13)
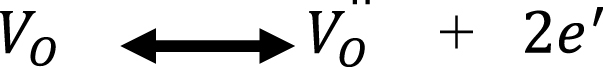


The conductivity of a material is mainly controlled by two factors: the content of charge carriers and the possibility of transporting them inside the material [100]. The formation of oxygen vacancies (OVs) in oxide materials is very easy by losing oxygen from the crystal lattice during heating at elevated temperatures [101]. The precise location of these electrons in these materials was still difficult to determine, as mentioned by *Ihrig* and *Hennings* [102]. However, it was very likely that these electrons clung to defects in oxygen, forming color centers, that could easily be thermally activated and transformed into conducting electrons (carriers) [103]. On the other hand, the decrease in AC conductivity for the two Y^3+^ doped compounds (BNYN0.02 and BNYN0.04), as shown in Fig. 15(d), is a logical consequence of the decrease in oxygen vacancy mobility in these compounds, as previously mentioned.

## 4 Conclusion

A general study of the structural, dielectric, and electrical properties of Ba_2_Na_(1-3x)_Y_x_Nb_5_O_15_ solid solution compositions (0 ≤ x ≤ 0.04) synthesized by the conventional solid-state method was performed. The X-ray diffraction study confirmed that the structure of all samples synthesized at 1200 °C is tungsten bronze with an orthorhombic space group *Cmm*2. The lattice parameters for the studied ceramics rank such that c<a<b indicating the crystallographic axes rotate 45° around the c axis at the tetragonal-orthorhombic transition. The SEM images showed high densification, low porosity, and a homogeneous microstructure consisting of regular grain size. The average grain size was between 4.807 and 5.267 μm. The largest value of the average grain size (D = 5.267 μm) was noticed for the compound BNYN0.02 which explains the volume reduction of this compound. The evolution of permittivity, and losses as a function of temperature (40 °C–600 °C) and 10 Hz-1 MHz frequency of the studied ceramics, shows that there are two phase transitions for the undoped BNN compound: the first one at about T_d_ = 350 °C associated with the symmetry change of the material (ferroelastic (*Cmm*2) to ferroelectric (*P*4*bm*)) and the second one at about T_m_ = 571 °C corresponding to another symmetry change (ferroelectric (*P*4*mm*) to paraelectric (*P*4/*mbm*)). The partial substitution of Na^+^ by a high valence ion Y^3+^ leads to a decrease of T_m_ and T_d_. The introduction of Y_2_O_3_ in the Ba_2_NaNb_5_O_15_ structure has a significant effect on the electrical conductivity and the resistance of the grains. The study of Nyquist diagrams of all ceramics confirms the semiconducting nature of our samples and the conduction process is thermally activated in these ceramics. The resistivity ρ of the prepared ceramics decreases with increasing temperature, validating the NTCR and indicating semiconductor-like behavior for all prepared samples. The Joncher power law fits the experimental AC conductivity data. The obtained ''n'' parameter values suggest that the small polaron non-overlapping tunneling effect (NSPT) is the appropriate model for understanding the charge transport mechanism in the BNN base compound, and for Y-doped BNN compounds, the Correlated Barrier Hopping (CBH) mechanism is the appropriate model. The obtained conduction activation energy values indicate that single and double-ionized oxygen vacancies are responsible for electrical conduction in Ba_2_Na_(1-3x)_Y_x_Nb_5_O_15_ ceramics.

## Data availability statement

Data will be made available on request.

## CRediT authorship contribution statement

**El Hassan Yahakoub:** Writing – original draft, Writing – review & editing. **Amine Bendahhou:** Investigation. **Ilyas Jalafi:** Methodology. **Fatima Chaou:** Resources. **Soufian EL Barkany:** Formal analysis, Software. **Zahra Bahari:** Project administration, Validation. **Mohamed Abou-Salama:** Supervision, Validation, Writing – review & editing.

## Declaration of competing interest

The authors declare that they have no known competing financial interests or personal relationships that could have appeared to influence the work reported in this paper.
